# A Cross-Sectional Survey Evaluating the Knowledge of Orthopaedic Surgeons in Intraoperative Radiation Safety Precautions

**DOI:** 10.7759/cureus.97086

**Published:** 2025-11-17

**Authors:** Khimi Karavadra, Swechhya Banstola, Neil Ashwood, Claire Gill, Muhammad Ali Fazal, Andrew Lacon

**Affiliations:** 1 Trauma and Orthopaedics, University Hospitals of Derby and Burton, Burton-upon-Trent, GBR; 2 Internal Medicine, Addenbrooke's Hospital, Cambridge, GBR; 3 Radiography, University Hospitals of Derby and Burton, Burton-upon-Trent, GBR; 4 Orthopaedics, Royal Free London NHS Foundation Trust, London, GBR; 5 Orthopaedics, University Hospitals of Derby and Burton, Burton-upon-Trent, GBR

**Keywords:** clinical competence, health care survey, ionising radiation exposure, orthopaedic surgery, #radiation protection, radiation safety and protection, united kingdom

## Abstract

Background

Intraoperative radiation has provided significant benefits to orthopaedic surgery, improving the accuracy of procedures and patient outcomes. However, the potential risks of ionising radiation cannot be overlooked. Guidelines and regulations such as Ionising Radiation (Medical Exposure) Regulations 2017 (IRMER), the As Low As Reasonably Achievable (ALARA), and the Radiation in Orthopaedics (RIO) study have established frameworks for minimising exposure while maximising the clinical effectiveness of intraoperative imaging. Ongoing training, technological advancements, and adherence to safety protocols are essential to ensure that the benefits of intraoperative radiation are achieved without compromising patient and clinician health and safety.

Methods

A 12-item questionnaire was prepared and initially piloted before being distributed to orthopaedic surgeons working within one trust. The survey assessed surgeon knowledge of existing radiation safety training, including trust policies and effects of radiation exposure, and perceptions on developing a training programme targeting intraoperative radiation safety.

Results

A total of 85 responses were collected, with a response rate of 65.9%, from an eligible sample of 129 orthopaedic surgeons. Of the participants, 44 (51.8%), 27 (31.8%), 10 (11.8%), and 4 (4.7%) were orthopaedic registrars, consultants, core trainees, and associate specialist doctors, respectively. Furthermore, 39 (45.9%) out of 85 surveyed stated they had received formal training in the use of fluoroscopy. Only 45 (52.9%) out of 85 surgeons were aware of radiation safety training in the trust. However, 75 (88.2%) out of 85 surgeons stated Yesto being worried about the effects of radiation on their health, and 83 out of 85 (97.6%) doctors surveyed responded Yes to supporting mandatory training in safe use of radiation in the operating room.

Conclusion

Despite existing guidance and health concerns regarding radiation exposure, orthopaedic surgeons in this study had gaps in such knowledge, potentially compromising health and safety precautions. A training package may improve knowledge of radiation safety for surgeons and operating room personnel.

## Introduction

Intraoperative radiation has provided expansive benefits to trauma and orthopaedic surgery since the introduction of the first C-arm in 1955 [1]. Such development has provided benefit to trauma and orthopaedic surgery, improving patient outcomes including better confirmation of alignment and encouragement of more minimally invasive procedures [1]. Although these benefits have been demonstrated, healthcare workers are exposed to occupational radiation through leakage of primary X-ray beams or scattering of unabsorbed X-rays [2]. While increasing doses of exposure provide a greater risk of recognised side effects of radiation, including carcinogenesis, hair loss, burns, and cataracts, stochastic effects can also occur, where such effects can result randomly with no apparent threshold of exposure [3].

Furthermore, a study of 1,203 female surgeons, comprising of 505 orthopaedic surgeons, observed two times greater prevalence of total cancer than expected [4]. This was 2.9 times greater than expected in the context of breast cancer [4].

In environments where occupational radiation exposure is prevalent, radiation safety regulations are designed to ensure comprehensive protection. Globally, radiation safety regulations are outlined by the International Commission on Radiological Protection (ICRP), providing a basis for the development of national regulatory frameworks [5].

## Materials and methods

**Figure 1:**
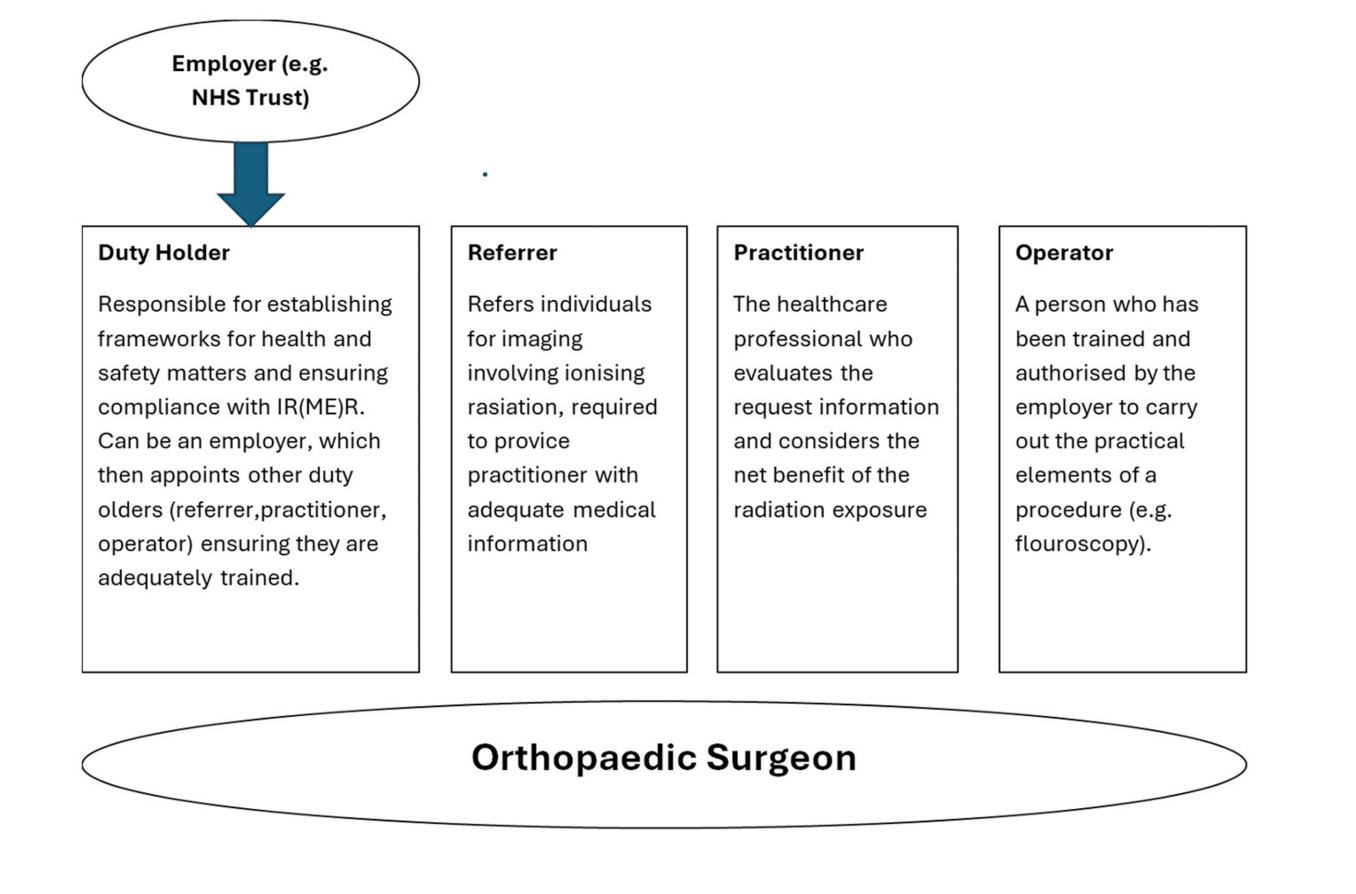
Roles within the IR(ME)R 2017 framework Adapted from the IR(ME)R framework [6] IR(ME)R, Ionising Radiation (Medical Exposure) Regulations

In the United Kingdom, Ionising Radiation (Medical Exposure) Regulations 2017 (IRMER) ensures safe, justified use of radiation, outlines radiation exposure limits in pregnancy, and provides guidance on sufficient personal protective equipment (PPE) [6]. The guidelines also delineate specific roles within radiological imaging, including the referrer, practitioner, and operator, each of whom may serve as a duty holder. In the context of orthopaedic surgery, the operating surgeon may hold responsibility for all four roles outlined in Figure 1 [6].

Orthopaedic surgeons have a responsibility to comply with the ALARA (As Low As Reasonably Achievable) principles and should have adequate training to ensure that they comply with the legislation and radiation safety principles such as ALARA [7,8].

Despite existing legislation and the integral role of the surgeon in procedures involving ionising radiation, there is still no mandatory requirement for formal training in radiation safety. This could have serious health consequences for all staff who work with ionising radiation in the orthopaedic department.

Aside from the patient, the operating surgeon receives the largest dose of radiation due to proximity to the source and scatter radiation from the patient. For example, during an intramedullary nailing procedure, the assistant would receive 37-62% of the dose received by the primary surgeon, with the scrub nurse receiving 11-20% [9].

Aim

This study aimed to assess existing knowledge of the local orthopaedic surgery team, including awareness of current radiation safety training and trust policies, knowledge of radiation exposure and PPE, and perceptions to outline a Training Needs Analysis for local training programme.

Objectives

The primary objective of this project was to evaluate the knowledge of orthopaedic surgeons in intraoperative radiation safety precautions. Secondary objectives further included identifying where gaps exist in such knowledge and assessing the need for a training programme to address these.

Methods

This study was undertaken within the Orthopaedic Department at the University Hospitals of Derby and Burton (UHDB), a secondary care setting providing elective and 24-hour acute orthopaedic care.

A survey of a mix of 12 multiple-choice questions, Yes/No questions, and short answer questions (Appendix) was initially curated. The questions were developed based on a previous survey and highlighted topics from the ALARA and Radiation in Orthopaedics (RIO) study [7,8,10]. The survey was piloted at the Royal Free Hospital, London, but the final data collection was conducted exclusively at the UHDB. Subsequently, the survey was distributed to all orthopaedic surgeons at UHDB through email, totalling 129 eligible orthopaedic doctors. The survey investigated four overarching themes: existing training on radiation safety/trust policy, knowledge of radiation safety and PPE, perspectives of own radiation safety, and attitudes to further training development.

As descriptive data was to be generated, further statistical analysis of p-values was not indicated.

Ethics

As the survey was part of a quality improvement project, the study did not require formal ethical approval.

## Results

Participant demographics

**Figure 2:**
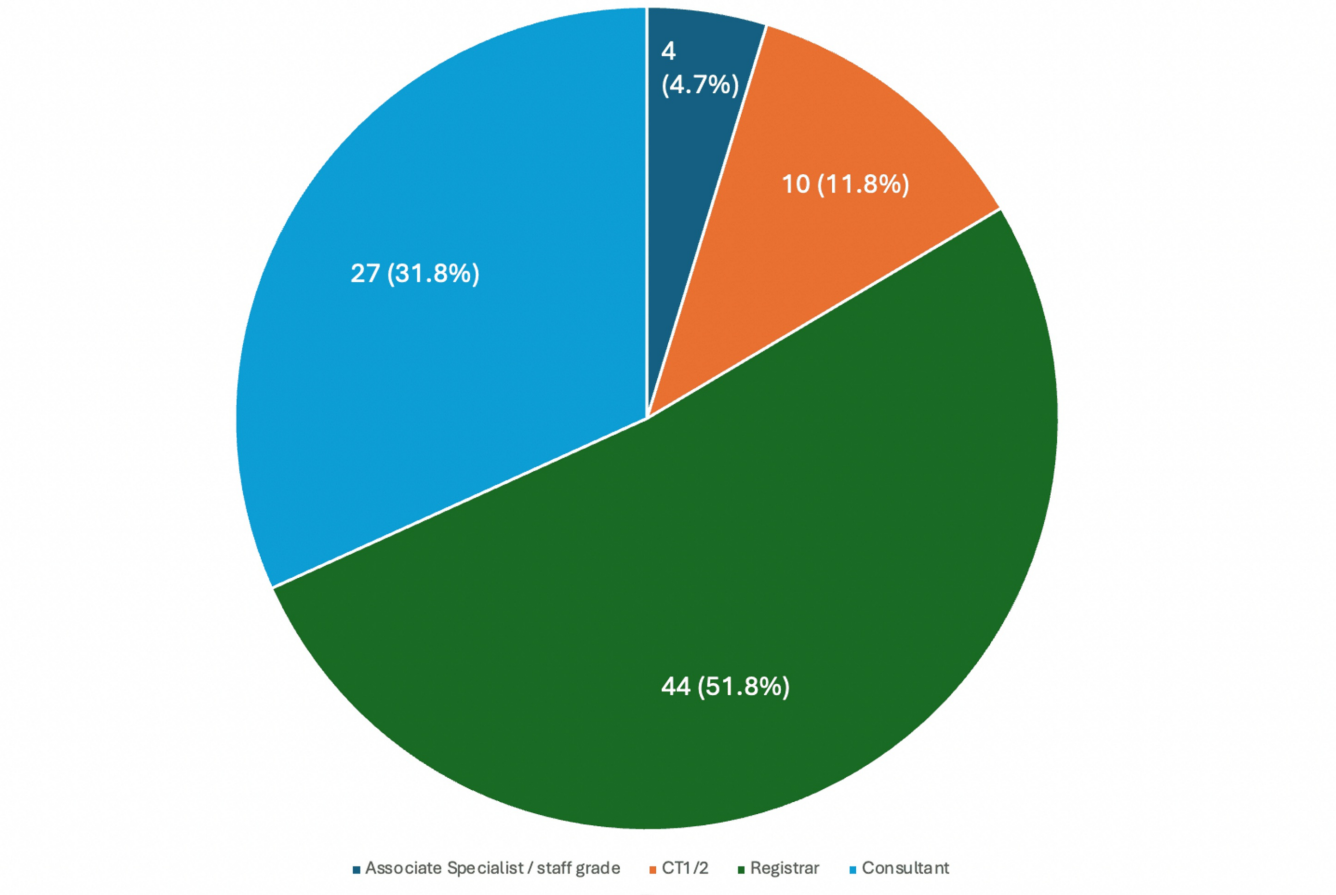
Training grades of surgeons participating in the study

A total of 85 responses were collected from a total eligible group of 129 orthopaedic doctors, with a response rate of 65.9%. All participants were at least two years post-graduation. Of these, 44 (51.8%) were orthopaedic registrars, 27 (31.8%) were consultants, 10 (11.8%) were core trainees, and 4 (4.7%) were associate specialist doctors (Figure 2).

Existing training on radiation safety/trust policy

Knowledge of existing training on radiation safety and trust policies was assessed using the questions: ‘Is there a compulsory radiation safety training in your trust?’, ‘Did you receive a formal training in safe use of fluoroscopy in operating theatres?’, ‘Do you know radiation protection supervisor of your trust?’ and ‘Are you aware of your trust’s policy of radiation protection for staff during pregnancy?’.

**Figure 3:**
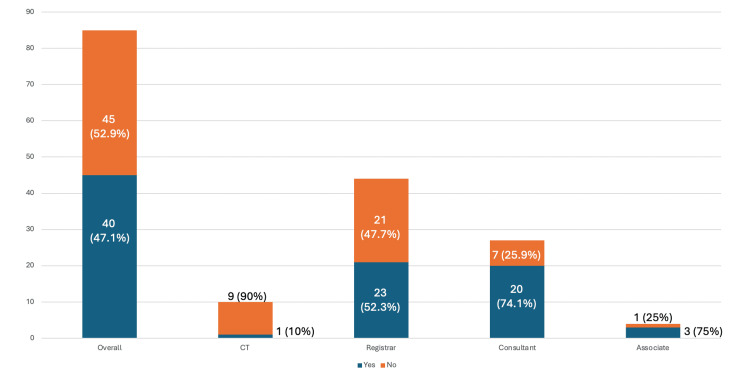
Compulsory training knowledge Is there compulsory radiation safety training in your trust?

Of the 85 participants, 45 (52.9%) reported Yes and 40 reported No to ‘Is there a compulsory radiation safety training in your trust?’ (Figure 3). Differing knowledge of existing compulsory radiation safety training was noted amongst specific groups of surgeons. Most consultants had knowledge of existing training, with 20 (74.1%) out of 27 answering Yes and subsequent 7 (25.9%) answering No. Similarly, three (75%) out of four Associate Specialist doctors answered Yes and one (25%) answered No. Registrars had a more even distribution of existing knowledge of training, with 21 (47.7%) out of 44 and 23 (52.3%) out of 44 answering Yes and No, respectively. However, amongst core trainee doctors, 9 (90%) out of 10 answered No, with only one (10%) answering Yes (Figure 3).

**Figure 4:**
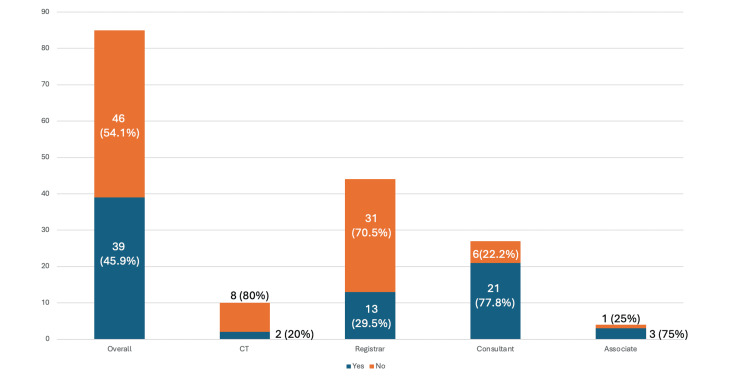
Survey of formal training in safe use of fluoroscopy Did you receive formal training in safe use of fluoroscopy in operating theatres?

When asked ‘Did you receive a formal training in safe use of fluoroscopy in operating theatres?’, 39 (45.9%) out of 85 total surveyed answered Yes and 46 (54.1%) answered No. Likewise, the distribution of this amongst each group of surgeons varied, with 21 (77.8%) out of 27 consultants and 3 (75%) out of 4 associate specialist doctors responding Yes. This contrasted to 13 (29.5%) out of 44 registrars and 2 (20%) out of 10 core trainees (Figure 4).

The question ‘Do you know radiation protection supervisor of your trust?’ was answered No by 70 (82.4%) out of 85 of those surveyed. Amongst each group, this consisted of 20 (74.1%) out of 27 of consultants, 2 (50%) out of 4 of associate specialist doctors, 38 (86.4%) out of 44 of registrars, and 10 (100%) out of 10 of core trainees.

**Figure 5:**
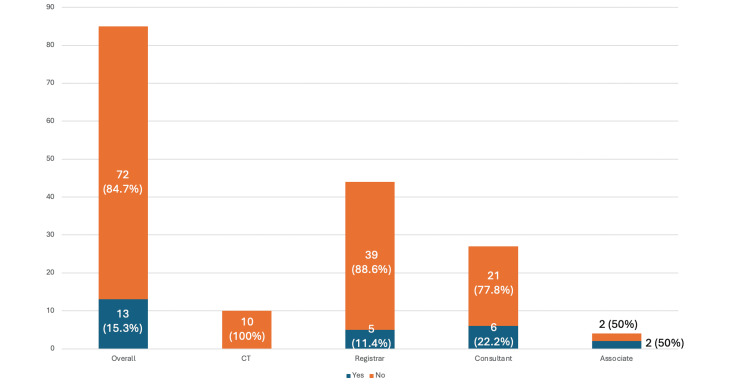
Awareness of trust policy for radiation protection for staff during pregnancy Are you aware of your trust's policy of radiation protection for staff during pregnancy?

Likewise, 72 (84.7%) out of 85 of all surveyed answered No to the question ‘Are you aware of your trust’s policy of radiation protection for staff during pregnancy?’. Within each group, this included 21 (77.8%) out of 27 of consultants, 2 (50%) out of 4 of associate specialist doctors, 39 (88.6%) out of 44 of registrars, and 10 (100%) out of 10 of core trainees (Figure 5).

Knowledge of radiation safety and PPE

Knowledge of Radiation Safety and PPE was assessed using the questions: ‘How would you rate your knowledge in the use of radiation in operating theatres?’, ‘Which of the following is the most radiosensitive tissue?’, ‘What information should be recorded at the time of the radiation exposure?’, ‘Do you know the annual radiation dose limit as recommended by the NCRP (National Council on Radiation Protection and Measurements)?’, and ‘When is there a requirement to wear lead protection (personal protective equipment)’.

**Figure 6:**
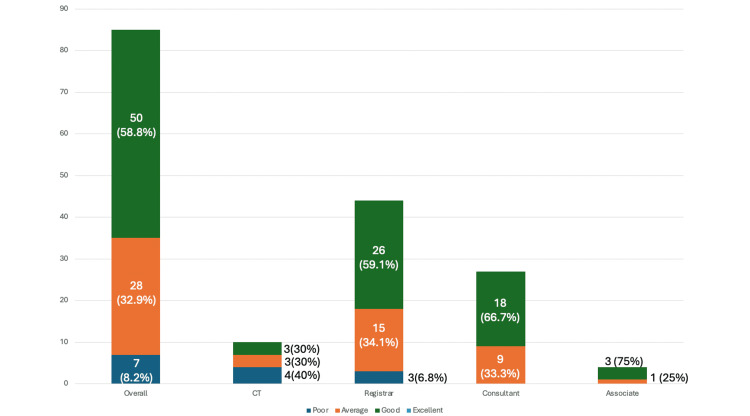
Knowledge of radiation safety How would you rate your knowledge in the use of radiation in operating theatres?

When asked to rate knowledge of use of radiation in operating theatres, of the 85 surgeons surveyed, 50 (58.8%) of 85, 28 (32.9%) of 85, and 7 (8.2%) of 85 self-rated knowledge to be good, average, and poor, respectively. No surgeons self-rated knowledge of such to be excellent. All associate specialist doctors and consultants reported their knowledge to be either good or average. This trend was also observed among 41 (93.2%) of 44 registrars and 6 (60%) of 10 core trainees, who similarly rated their knowledge within these categories (Figure 6).

The question ‘Which of the following is the most radiosensitive tissue?’ was answered correctly overall, with 32 (37.6%) of 85 of surgeons answering eye lens. This was correctly answered by 0 (0%) of 4 of associate specialist doctors, 5 (18.5%) of 27 of consultants, 3 (30%) of 10 of core trainees, and 24 54.5%) of 44 of registrars. Most surgeons submitted answering thyroid, with 49 (57.6%) out of 85.

Furthermore, 59 (69.4%) of 85 of surgeons reported not knowing the annual radiation dose limit as recommended by the NCRP (National Council on Radiation Protection and Measurements), this included 1 (25%) of 4 of associate specialist doctors, 17 (63.0%) of 27 consultants, 32 (72.7%) of 44 registrars, and 9 (90%) of 10 core trainees.

Perspectives of own radiation safety

**Figure 7:**
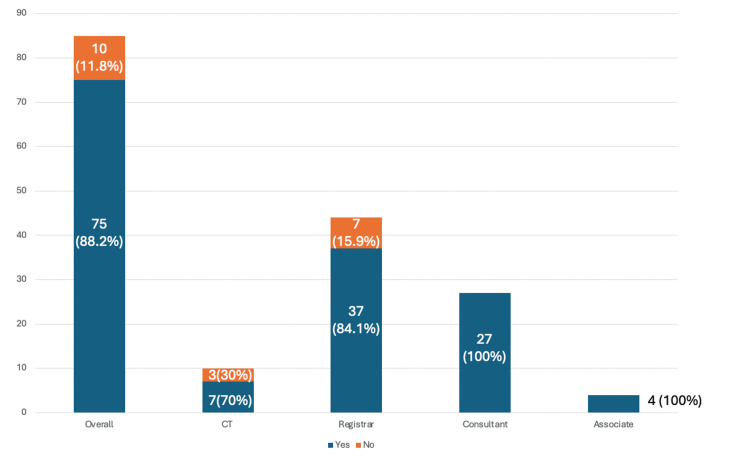
Health risks of radiation Are you worries about effects of radiation on your health?

The question ‘Are you worried about effects of radiation at work on your health?’ explored surgeon’s perspective of own radiation safety; 75 (88.2%) of 85 of all surgeons surveyed reported Yes. Of this, all consultants and specialist associate doctors, 37 (84.1%) out of 44 registrars, and 7 (70%) out of 10 of core trainee doctors answered Yes (Figure 7).

Attitudes to further training development

**Figure 8:**
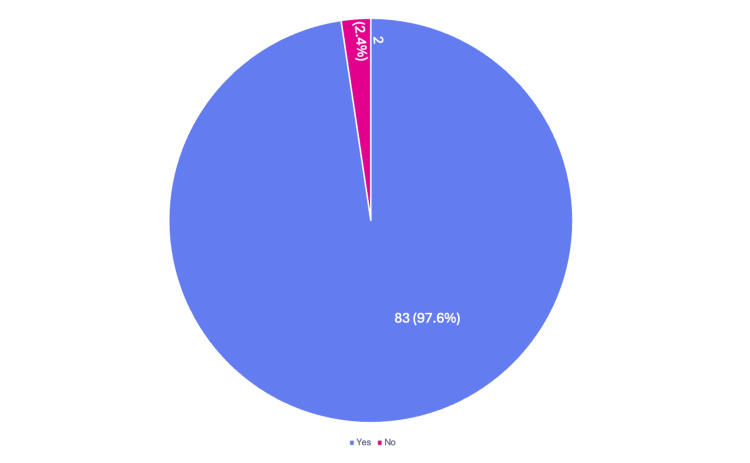
Support for radiation safety training Do you support mandatory training in safe use of radiation in operating theatres?

Out of 85 doctors surveyed, 83 (97.6%) responded Yes to supporting mandatory training in safe use of radiation in the operating room (Figure 8). Of the two (2.4%) doctors, who stated not supporting it, one was a core trainee doctor and one was a registrar. When asked how often the training should be, most doctors responded Yearly or Two Yearly, both preferred by 34 (40%) out of 85 of participants each, and Three Yearly, by 17 (20%) out of 85 of participants (Figure 8).

## Discussion

This survey highlights critical areas where orthopaedic surgeons demonstrate limited understanding of ionising radiation, including foundational concepts, relevant regulatory frameworks, and safe operational practices in the theatre setting. These deficiencies have important implications for both patient and staff safety and underscore the need for targeted educational interventions. Our findings are consistent with the literature, including regional surveys and the first national survey of 406 orthopaedic surgeons [10].

Although our study did not explore the explanations for lack of knowledge, it may be due to its voluntary nature. A lack of standardised training modules, time constraints with concurrent clinical commitments, and perceived low risk of radiation may be contributing factors. Further research is required and may be helpful when designing a training package to ensure training is effective.

The RIO study [10] was the first national survey in the UK to assess orthopaedic surgeons’ knowledge of radiation safety, ionising radiation principles, relevant legislation, and operative practices. Conducted four years ago, it identified significant gaps in knowledge and emphasised the need for a nationwide training program. With a considerably larger sample size of 406 surgeons, compared to our local trust-wide cohort of 85, the RIO study provides a valuable benchmark. Our findings are consistent with those of the RIO study, as we also identified a substantial proportion of surgeons who had received no formal training in radiation safety. Notably, the percentage in our study is higher, which may be attributable to the smaller sample size.

The survey outlined poor evidence of training, with 46 (54.1%) out of 85 surgeons not receiving formal training in fluoroscopy. Ranade et al. also found deficiencies in training with 92.4% of surgeons citing they had had no formal training [11]. Raza et al. found 38.1% of surgeons received no training [10], similar to Snowden et al., who found that 40 surgeons out of 77 (51.9%) had no formal training [12]. Currently formal instruction in radiation safety is not currently a mandatory part of orthopaedic training [8]. Contrastingly, a nationwide survey conducted in the United States indicated that approximately 80% of orthopaedic residents had received formal training in general radiation safety [13]. With nearly 40% of surgeons self-rating their knowledge of radiation average or poor, there is a need for a formal training package. Our findings support the existing literature, which calls for formal training in radiation safety.

Rowantree and Currie [2] suggest that formal and continuous training should be provided to ensure all staff are safe and aware of the risks of ionising radiation. This is supported by our data, suggesting that most surgeons wanted yearly or two-yearly training. However, there is a paucity of literature regarding how often training packages should be conducted and requires further research. Most surgeons in our survey (88.2%) were worried about the effects of radiation on their health, which is consistent with the findings of Fidan et al., who found that 87% of surgeons were concerned regarding radiation exposure in a survey of 180 surgeons [14].

Furthermore, in our survey, 84% of surgeons were unaware of the trust’s radiation safety officer in pregnancy, highlighting a clear gap in knowledge. Given evidence that 96% of pregnant female healthcare workers remain employed through at least the 16th week of gestation, the potential for occupational radiation exposure among orthopaedic surgeons during pregnancy warrants careful evaluation. Furthermore, previous studies have identified ionising radiation, such as that emitted from image intensifiers, as a risk factor for cancer, particularly among female surgeons, due to the radiosensitivity of breast tissue [15].

Additionally, the International Commission on Radiological Protection (ICRP) recommends that once a worker is aware of their pregnancy, the equivalent dose to the child in utero should not exceed 1 mGy for the remainder of the pregnancy [6]. In the UK, the Ionising Radiations Regulations (IRR) adopt these recommendations through legislation, stipulating that for female employees of reproductive capacity, the abdominal effective dose must not exceed 13 mSv over any consecutive three-month period. For clinical context, fluoroscopic procedures such as femoral nailing with distal locking are associated with an estimated foetal dose of approximately 0.044 mGy per procedure, indicating that up to 23 such procedures could theoretically be performed before reaching the recommended exposure threshold [15]. In contrast, procedures such as dynamic hip screw fixation deliver a substantially lower estimated foetal dose of approximately 0.001 mGy, permitting up to 800 procedures before approaching the same limit [15]. These findings highlight the importance of adherence to radiation protection protocols, use of PPE, and proactive occupational risk assessments for pregnant and reproductive-age healthcare professionals involved in fluoroscopic procedures.

Currently, only 7% of U.K. consultant orthopaedic surgeons are female [16]. However, the number of female orthopaedic trainees is increasing, reflecting a more inclusive and evolving workforce. The British Orthopaedic Association has established a ‘short working life group’ to gain a deeper understanding of the breast cancer risks in female orthopaedic surgeons [8]; it is a collective effort to ensure that all staff, including pregnant women, are safe and aware of the risks of ionising radiation and how to mitigate them.

Study limitations

We acknowledge that only surgeons’ responses from two trusts were used; hence, there could be selection bias and limited generalisability. Furthermore, there is potential response and recall bias due to voluntary participation and self-reported nature of this study. Data analysis methods included descriptive statistics and subgroup comparisons. Whilst there is limited statistical analysis and a smaller sample size, with a 65.9% response rate, we believe that this does not undermine the validity of our findings. It is further important to note that although the questionnaire used was not pre-validated, it was initially piloted to ensure readability.

## Conclusions

This study provides an insight into the knowledge of intraoperative radiation safety amongst orthopaedic surgeons and their perspectives to developing a training package. It is important to note that although the questions asked were developed by members of staff utilising intraoperative radiation, they do not represent a standardised set of questions on the topic. Although constructed locally, our findings did align with prior conclusions and, hence, support further need for the development of such training programmes.

Future research could consider greater qualitative research on the attitudes and behaviours of surgeons to radiation safety knowledge and use of PPE, with regular audits ensuring adequate radioprotection. Likewise, we suggest the development of a formal training package, with pre- and post-training analysis to objectively determine the efficacy of such a programme.

## Appendices

Radiation Survey

Dear Colleagues

We are conducting a survey on radiation awareness among trauma and orthopedic surgeons and request your participation in this evaluation which will involve filling in following brief questionnaire. It should take no more than 10 minutes of your precious time. It is an anonymous survey and will be used for quality improvement and research.

Please circle or tick the appropriate: ◯ Consultant      ⃝ Registrar     ⃝ Associate Specialist/Staff Grade 

 ⃝ CT1/2

1.     Is there a compulsory radiation safety training in your trust?                

      Yes                                                      No

2.     Did you receive a formal training in safe use of fluoroscopy in operating theaters?

      Yes                                                      No

3.     Do you know radiation protection supervisor of your trust?                              

       Yes                                                      No

4.     Are you aware of your trust’s policy of radiation protection for staff during pregnancy?

        Yes                                                      No

5.     How would you rate your knowledge in the use of radiation in operating theatre?

Excellent                                       Good                                       Average                                       Poor

6.     Which of the following is the most radiosensitive tissue?

Finger                                       Eye lens                                  Thyroid

7.     What information should be recorded at the time of the radiation exposure?

8.     Do you know the annual radiation dose limit as recommended by the NCRP (National Council on Radiation Protection and Measurements)?                            

         Yes                                                      No

9.     Are you worried about effects of radiation at work on your health?                  

         Yes                                                       No

10.     When is there a requirement to wear lead protection (personal protective equipment)

11.     Do you support mandatory training in safe use of radiation in operating room?

          Yes                                                      No

12.     How often the training should be

          Yearly                                    Two yearly                                   Three yearly

Many thanks for completing the survey.
